# Dietary Changes Are Associated with Seasonal Restructuring of the Gut Microbiome in *Cervus nippon kopschi*

**DOI:** 10.3390/microorganisms14030674

**Published:** 2026-03-16

**Authors:** Yang Zhang, Tianxiang Zhang, Manyu Zhang, Yumeng Jia, Xiaofeng Huang

**Affiliations:** 1 Jiangxi Academy of Forestry, Nanchang 330013, China; yangzhang3217@163.com (Y.Z.); toughtom@163.com (T.Z.); manyu_zhang@126.com (M.Z.); 2 College of Forestry, Jiangxi Agricultural University, Nanchang 330045, China; jiayumeng990310@163.com

**Keywords:** *Cervus nippon kopschi*, gut microbiome, dietary, seasonal shifts, DNA metabarcoding, 16S rRNA sequencing

## Abstract

Seasonal dietary shifts are associated with significant alterations in the gut microbiome of herbivores, yet the specific impacts of these shifts on microbial metabolic functions have not been fully elucidated. To address this gap, we employed DNA metabarcoding of fecal samples and 16S rRNA gene sequencing to explore the relationship between seasonal diet and gut microbiome composition in a population of sika deer (*Cervus nippon kopschi*). Our findings indicate pronounced seasonal variations in both dietary composition and gut microbial community structure. Notably, during the winter months, the gut microbiome exhibited a significant enrichment of predicted pathways (predicted using PICRUSt2) related to fatty acid and lipid biosynthesis and degradation, amino acid degradation, and the TCA cycle. Conversely, the active growing seasons (spring and summer) were characterized by enhanced glycolysis and amino acid biosynthesis pathways. These functional shifts showed significant correlations with seasonal changes in dietary nutrients, such as crude protein and fiber, and climatic factors. Our results suggest that seasonal dietary changes are associated with a restructuring of the gut microbiome’s metabolic potential, which may assist sika deer in adapting to fluctuating physiological demands and environmental challenges across different seasons.

## 1. Introduction

The gut microbiome, a complex and dynamic ecosystem of microorganisms located within the gastrointestinal tract [1], has increasingly been recognized as a crucial element of host biology [2], health [3], and evolutionary processes [4]. This intricate community, comprising bacteria, archaea, fungi, and viruses, establishes a symbiotic relationship with its host [5,6,7], performing essential functions that the host genome cannot fulfill independently [8,9]. The composition and functional capabilities of this microbial consortium are influenced by host-intrinsic factors and environmental variables [10].

Among environmental factors, diet profoundly influences the structure and function of the gut microbiome [11]. Dietary substrates directly affect the selection of microbial taxa, leading to rapid shifts in community composition, a phenomenon that is well-documented in humans. However, to gain a comprehensive understanding of the ecological and evolutionary dynamics between hosts and microbes, research must expand to include wild animal populations. Unlike humans or animals in captivity, wild animals are subjected to natural selective pressures, such as fluctuating food availability, predation, and climatic variability [10]. Investigations into the gut microbiomes of wild animals can therefore provide insights into microbial adaptive plasticity and their role in host adaptation to challenging environments.

Seasonality represents a significant and predictable form of environmental change that profoundly influences the physiology and behavior of wildlife, especially in temperate and sub-tropical ecosystems. Seasonal variations in temperature, precipitation, and photoperiod regulate the life cycles of both plants and animals, leading to substantial shifts in resource availability and quality [12]. As a result, many animals are compelled to modify their foraging strategies, diets, and metabolic processes to ensure survival and reproductive success throughout the year. Consequently, these seasonal dietary changes are expected to induce corresponding cyclical modifications in the gut microbiome. Indeed, an expanding corpus of research has documented seasonal variations in the gut microbiota across a diverse array of taxa, including humans [11,13], non-human primates [14,15,16], large herbivores [10], giant pandas [17], birds [12,18], and even insects [19]. These studies collectively illustrate that the gut microbiome is not a static entity but rather a responsive and adaptable community that aligns closely with environmental and dietary transitions.

Herbivorous mammals present a unique opportunity for investigating the seasonal dynamics of the diet-microbiome axis due to their reliance on plant matter, which is rich in complex carbohydrates. This reliance necessitates a symbiotic relationship with gut microbes to facilitate energy extraction [10]. The nutritional landscape these mammals encounter varies significantly with the seasons. During the growing season, they have access to abundant, nutrient-dense forage such as young leaves, shoots, and flowers, which are high in protein and digestible carbohydrates [17]. In contrast, the dormant season is characterized by a scarcity of food, forcing these animals to depend on lower-quality, high-fiber items like mature leaves, twigs, and bark [20]. To adapt, herbivores require a flexible digestive physiology, primarily driven by the adaptive capacity of their gut microbiome. The microbial community’s ability to reconfigure its composition and functional repertoire is crucial for maximizing nutrient extraction and ensuring host survival.

The South China sika deer (*Cervus nippon kopschi*), an endangered subspecies native to the mid-lower Yangtze River basin, serves as an excellent model for exploring these seasonal adaptations [21]. This region features distinct seasons that provide the deer with a cyclically changing environment and food supply. Previous DNA metabarcoding studies have characterized the broad, seasonally shifting diet of this subspecies, identifying dominant plant families such as Rosaceae and noting variations between summer and winter [22,23,24]. Further investigations have linked these dietary patterns to changes in the gut microbiome, demonstrating that sika deer can flexibly reshape their microbial communities in response to seasonal changes in diet and nutrient availability [20].

Despite the established correlation between season, diet, and gut microbial composition in *C. n. kopschi*, a comprehensive, year-round investigation that deeply integrates dietary composition with the microbiome’s functional metabolic adaptations remains necessary. Most studies have either focused on taxonomic shifts or compared only two seasons, which leaves the nuanced transitions across all four seasons and the specific metabolic pathways underpinning these adaptations less explored. It is still unclear how the gut microbiome’s functional capacity is reorganized throughout the year to accommodate vastly different nutritional inputs.

In this year-long study, we investigated a wild population of South China sika deer using DNA metabarcoding and 16S rRNA gene sequencing to characterize their diet and gut microbiome across all four seasons. Our objective was to elucidate the functional consequences of seasonal microbiome shifts, hypothesizing that distinct seasonal patterns in microbiome composition and functional potential would mirror dietary changes. We predicted that metabolic pathways would dynamically reconfigure to optimize digestion, potentially enhancing energy capture from simple carbohydrates during the growing seasons (spring and summer) and shifting towards the breakdown of complex fibers during the nutrient-poor dormant season (winter). This study aims to provide a comprehensive understanding of these predicted functional shifts to inform conservation and management strategies for this endangered species.

## 2. Materials and Methods

### 2.1. Fecal Sample Collection

**Figure 1:**
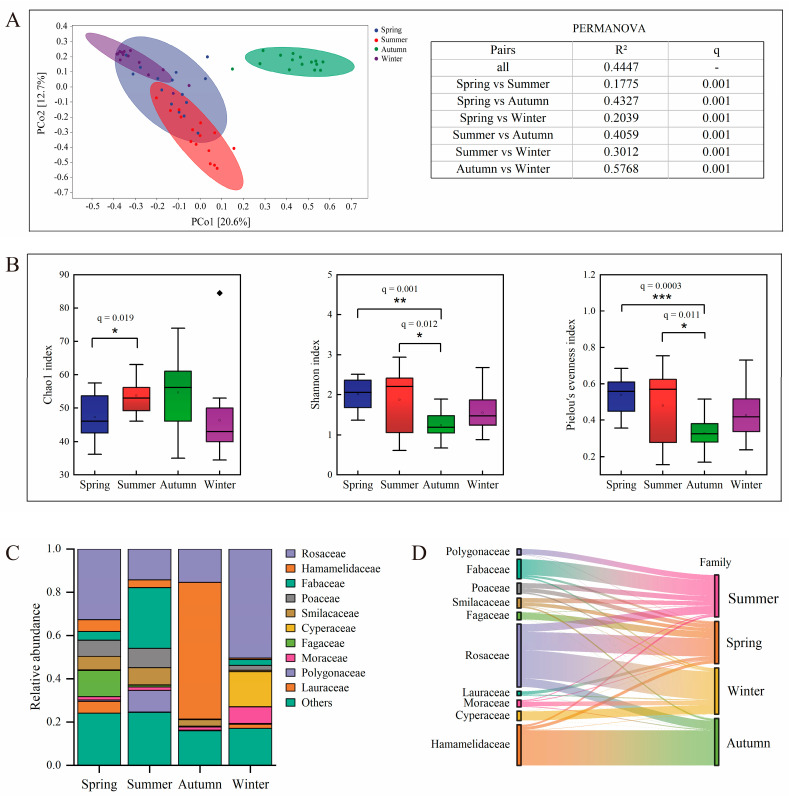
Seasonal variation in the dietary composition and diversity of sika deer: (**A**) Principal coordinate analysis (PCoA) of ASV relative abundances, using Bray–Curtis distances, was performed to visualize compositional differences; each circle represents an individual sample; different colors represent different experimental groups as indicated in the legend. PERMANOVA was applied to test for significant differences in dietary composition among seasons. (**B**) Boxplot of the Chao1 index, Shannon index, and Pielou’s evenness index. The boxplot shows the within–group distribution of the samples from different seasons (medians/IQR); data points falling outside 1.5 times the IQR are defined as outliers and are represented by diamonds. (**C**) Bar chart of seasonal differences in the relative abundance of major families. (**D**) A Sankey diagram was used to track indicator families associated with each season; lines indicate the relationship between indicator families and seasons, with colors labeled according to plant families; the line width is scaled to reflect the indicator value. Adjusted *p*–values (q) < 0.05 were considered significant and are shown as * q < 0.05, ** q < 0.01, *** q < 0.001.

Sampling was conducted over four seasons from October 2022 to July 2023. Fecal samples from wild sika deer were collected during winter (January, *n* = 15), spring (April, *n* = 15), summer (July, *n* = 15), and autumn (October, *n* = 15) at the Jiangxi Taohongling Sika Deer National Nature Reserve, located in Pengze County, Jiujiang City, Jiangxi Province (116°32′~116°43′ E; 29°42′~29°53′ N) (Figure 1). To minimize the likelihood of resampling the same individual, only fresh fecal samples characterized by moist, warm, and glossy surfaces were selected. Pellets from the same defecation event were avoided, and a minimum distance of 100 m between consecutive sampling points was maintained during each field survey, as terrain and access permitted. Sampling was conducted across multiple days, seasons, and locations, with the GPS coordinates, date, and time of each collection documented. Samplers wore sterile disposable gloves and utilized sterile cotton swabs to transfer the fresh fecal samples into 50 mL sterile centrifuge tubes. The samples were immediately frozen in liquid nitrogen, transported to the laboratory using dry ice, and stored at −80 °C in an ultra-low temperature freezer for subsequent DNA extraction.

### 2.2. DNA Extraction

Total microbial and plant DNA was extracted from all fecal samples using the MiniBEST Universal Genomic DNA Extraction Kit (TaKaRa, Tokyo, Japan) following the manufacturer’s instructions. The DNA extracts were evaluated by 1% agarose gel electrophoresis and quantified using a NanoDrop 2000 UV-Vis Spectrophotometer (Thermo Scientific, Wilmington, DE, USA).

### 2.3. 16S rRNA Gene Amplicon Sequencing

PCR amplification of the bacterial 16S rRNA genes from the V3-V4 regions was conducted using the forward primer 338F (5′-ACTCCTACGGGAGGCAGCA-3′) and the reverse primer 806R (5′-GGACTACHVGGGTWTCTAAT-3′). Sample-specific 7 bp barcodes were incorporated into the primers to facilitate multiplex sequencing. The PCR mixture contained 5 μL of buffer (5×), 0.25 μL of Fast pfu DNA Polymerase (5 U/μL), 2 μL (2.5 mM) of dNTPs, 1 μL (10 μM) of each Forward and Reverse primer, 1 μL of DNA template, and 14.75 μL of ddH_2_O. Thermal cycling consisted of initial denaturation at 98 °C for 5 min, followed by 35 cycles of denaturation at 98 °C for 30 s, annealing at 53 °C for 30 s, and extension at 72 °C for 45 s, with a final extension at 72 °C for 5 min. PCR amplicons were purified using Vazyme VAHTSTM DNA Clean Beads (Vazyme, Nanjing, China) and quantified with the Quant-iT PicoGreen dsDNA Assay Kit (Invitrogen, Carlsbad, CA, USA). After the individual quantification, amplicons were pooled in equal amounts, and pair-end 2 × 250 bp sequencing was performed using the Illumina NovaSeq platform with the NovaSeq 6000 SP Reagent Kit (Illumina, San Diego, CA, USA) (500 cycles).

### 2.4. Diet DNA Metabarcoding

For DNA metabarcoding, the chloroplast *rbc*L region was utilized, employing the forward primer *rbc*L-F (5′-CTTACCAGYCTTGATCGTTACAAAGG-3′) and the reverse primer *rbc*L-R (5′-GTAAAATCAAGTCCACCRCG-3′). Sample-specific 7 bp barcodes were integrated into the primers to facilitate multiplex sequencing. The PCR assays comprised 25 μL reactions containing 5 μL of buffer (5×), 0.25 μL of Fast pfu DNA Polymerase (5 U/μL), 2 μL of dNTPs (2.5 mM), 2 μL of primers (10 μM), 1 μL of DNA template, and 14.75 μL of ddH_2_O. The thermocycling protocol included an initial denaturation at 98 °C for 5 min, followed by 35 cycles of 98 °C for 30 s, 55 °C for 30 s, and 72 °C for 1 min, with a final extension at 72 °C for 5 min. A negative control was employed to evaluate the impact of laboratory procedures on the outcomes. Sequencing was performed on the Illumina NovaSeq platform.

### 2.5. Reference Plant DNA Libraries

To identify plant sequences in the diets from fecal samples, a comprehensive DNA reference database was established, encompassing plant species within the Jiangxi Taohongling Sika Deer National Nature Reserve. This database includes the 232 most abundant species in the area. All plant species were identified by professional botanists. Reference plant DNA was extracted using the MiniBEST Universal Genomic DNA Extraction Kit (TaKaRa, Tokyo, Japan) from 0.2 g of leaves, and the chloroplast *rbc*L gene was amplified using established primers and protocols. The PCR products were verified by 1% agarose gel electrophoresis, sequenced using an ABI 3730xl analyzer (Applied Biosystems, Foster City, CA, USA), and the sequences were assembled using DNAman (v8.0).

### 2.6. DNA Metabarcoding Sequence Analysis

Bioinformatic analyses of the diet were conducted using QIIME2 (v2019.4). Initially, raw sequence data were demultiplexed using the demux plugin, and the primers were trimmed using the cutadapt plugin [25]. Following this, sequences were quality filtered, denoised, merged, and chimeras were removed using the DADA2 plugin [26]. The resulting sequences were merged based on 100% similarity to generate feature sequences, Amplicon Sequence Variants (ASVs), and abundance data tables. These ASV feature sequences were then compared against the reference plant DNA libraries to ascertain the taxonomic information for each ASV. The principles for species annotation are as follows: (1) If sequence identity is ≥99% and corresponds to a single species, annotation is assigned to that species. (2) If sequence identity is ≥99% and corresponds to multiple species, annotation is assigned to the lowest taxonomic unit that encompasses all identified species. (3) If sequence similarity is between 97% and 99% and corresponds to a single species, annotation is assigned to the species’s immediate higher taxonomic unit. (4) If sequence similarity is between 97% and 99% and corresponds to multiple species, annotation is assigned to the lowest taxonomic unit that encompasses all identified species.

### 2.7. Microbiota Community Analysis

Microbiome bioinformatics analyses were performed using QIIME2 (v2019.4). Raw sequence data were initially demultiplexed using the demux plugin, followed by primer trimming with the cutadapt plugin [25]. The sequences were subsequently quality filtered, de-noised, merged, and chimera-removed using the DADA2 plugin [26]. To ensure reproducibility, specific parameters were set in DADA2: truncation lengths were established at --p-trunc-len-f 223 and --p-trunc-len-r 229; quality filtering employed maximum expected error thresholds of --p-max-ee-f 2 for forward reads and --p-max-ee-r 4 for reverse reads; and chimera removal utilized the minimum fold parent overabundance method (--p-min-fold-parent-over-abundance). The resulting sequences were merged at 100% similarity to generate amplicon sequence variants (ASVs) and the corresponding feature abundance table. Read depths per sample before and after filtering were extracted from the DADA2 summary outputs (columns “input” for pre-filtering and “denoised” for post-filtering) and were used to report sample retention and data loss during processing. For direct comparison with previous studies of sika deer and to ensure downstream PICRUSt2 (v2.5.2) compatibility, ASV taxonomic assignment was conducted against the Greengenes (v13_8) reference database. Representative ASV sequences were classified in QIIME2 (v2019.4) using the feature-classifier plugin’s classify-sklearn method with a pre-trained Naive Bayes classifier based on Greengenes (v13_8). The classify-sklearn was executed using QIIME2’s default parameters, with a confidence threshold for taxonomic assignment set to e < 1 × 10^−5^.

### 2.8. Environmental Factors and Chemical Composition Analysis

Air temperature (quarterly mean temperature) and precipitation (quarterly precipitation) at the sampling sites were obtained from the National Meteorological Administration of China. Based on the results of a seasonal dietary analysis, the top ten plants in relative abundance for each season were collected and brought back to the laboratory. The top ten plant leaves consumed by sika deer in each season were taken back to the laboratory and dried to a constant weight at 60 °C. The dried samples were then ground and passed through a 0.25 mm sieve for chemical composition analysis. Crude protein was determined by the Kjeldahl method [27], crude fat by the Soxhlet extraction method [27], crude fiber according to Van Soest et al. [28], and P and Ca by inductively coupled plasma mass spectrometry [29].

### 2.9. Statistical Analysis

Sequence data analyses were conducted using QIIME2 (v2019.4) and R packages (v4.3.3). ASV-level alpha diversity was calculated with the ASV table in QIIME2 (v2019.4) and depicted in box plots. Beta diversity analysis was undertaken to explore the structural variation in microbial and plant communities across samples using Bray–Curtis distances [30]. A principal coordinate analysis (PCoA) was performed employing the “vegan” and “ade4” packages [31]. Heatmaps were generated using the R package “pheatmap”. Differences in the microbiota and diet structure among groups were assessed using permutational multivariate analysis of variance (PERMANOVA) with the “adonis2” function in the “vegan” package, employing 999 permutations [32]. Prior to conducting PERMANOVA, the homogeneity of multivariate dispersion among groups was evaluated using “betadisper” (vegan) and assessed by a permutational test (permutest) with 999 permutations; results from PERMANOVA were interpreted as indicative of differences in group centroids only when dispersion differences were not significant (*p* > 0.05). Taxonomy compositions and abundances were visualized using MEGAN (v7.0) [33] and GraPhlAn (v1.1.3) [34]. Redundancy analysis (RDA) and variance partitioning analysis (VPA) based on a distance-based linear model were utilized to quantify the impacts of external factors (such as temperature, precipitation, and five dietary components) on the gut microbiota. A Mantel test was applied to analyze the correlation between the matrices. ASV sequences were aligned to the reference 16S rRNA gene phylogeny using PICRUSt2’s placement pipeline (EPA-NG/gappa), which facilitated the generation of a combined reference-plus-query tree. Subsequently, gene-family copy numbers for each ASV were estimated using the Castor hidden-state prediction (HSP) algorithm; PICRUSt2 (v2.5.2) calculated per-sequence and per-sample NSTI values, excluding sequences with NSTI values greater than 2 from downstream analyses. Predicted gene-family counts were normalized by 16S rRNA gene copy number (normalize_by_copy_number) and integrated with observed ASV abundances to determine per-sample predicted gene-family abundances. Gene-family abundances were then mapped to MetaCyc pathways using PICRUSt2’s default mapping and MinPath inference, producing predicted MetaCyc pathway abundances for each sample. To identify MetaCyc pathways significantly enriched across seasons, we used MaAsLin3 (v1.3.0) with season as the fixed effect. Pathway relative abundances were normalized by Total Sum Scaling (TSS) followed by LOG transformation. Features were filtered using a minimum prevalence of 0.1 and a minimum abundance of 0.001, and the augment = TRUE parameter was enabled for model robustness.

For comparisons among multiple groups (including diversity indices, environmental variables, and functional pathway abundances), differences were initially tested using the nonparametric Kruskal–Wallis test. When this test indicated a significant overall effect (*p* < 0.05), pairwise post hoc comparisons were conducted using Dunn’s test. To manage the false discovery rate associated with multiple comparisons, *p*-values from these tests (including post hoc pairwise comparisons and other multiple hypothesis tests) were adjusted using the Benjamini–Hochberg procedure. Adjusted *p*-values (q) less than 0.05 were considered statistically significant and are denoted as * q < 0.05, ** q < 0.01, *** q < 0.001.

## 3. Results

### 3.1. Diet Diversity and Composition Across Seasons

The dietary data comprised 5,962,315 high-quality sequences, 5,961,708 non-singleton sequences, and 1938 ASVs (Table S1). These sequences represented 57 plant families, 76 genera, and 79 species. The dietary composition showed significant seasonal variations. PERMANOVA (adonis2, vegan; 999 permutations) confirmed that the dietary composition differed significantly across all seasonal pairs (q = 0.001), with the greatest dissimilarity observed between autumn and winter (R^2^ = 0.5768) (Figure 1A, Table S2). This distinct seasonal structuring was visually supported by PCoA ordination, where confidence ellipses for the summer, autumn, and winter samples were clearly separated (Figure 1A). Although the spring ellipse partially overlapped with those of summer and winter, its unique centroid position confirmed its distinct dietary profile.

Seasonal variation in dietary alpha diversity was apparent across all metrics (Figure 1, Table S3). The Chao1 index demonstrated significantly higher species richness during summer compared to spring (q = 0.019), while no significant differences were observed between other seasonal comparisons. Conversely, both the Shannon and Pielou indices indicated that dietary evenness was markedly higher in spring and summer than in autumn, with these differences being statistically significant (Shannon: q = 0.001, 0.012; Pielou: q = 0.0003, 0.011). No significant differences in dietary evenness were found between spring and summer, or between either of these seasons and winter. These patterns suggest that summer exhibited the peak season for dietary species richness, whereas spring and summer were characterized by more balanced use of dietary resources, and autumn showed reduced dietary evenness.

At the family level, core dietary components exhibited marked shifts (Figure 1C, Table S4). In spring, Rosaceae and Fagaceae were predominant, along with Poaceae. The summer diet primarily consisted of Fagaceae, Rosaceae, and Polygonaceae. Autumn displayed a distinct shift toward Hamamelidaceae, Rosaceae, and Smilacaceae, while winter diets predominantly returned to Rosaceae, accompanied by Cyperaceae and Moraceae. As indicator plant species, Rosaceae was prevalent in spring and winter, Hamamelidaceae in autumn, and Fabaceae in summer (Figure 1D). The metabarcoding analysis demonstrates that sika deer exhibit pronounced seasonal plasticity in their foraging strategies.

### 3.2. Gut Microbiota Diversity and Composition Across Seasons

**Figure 2:**
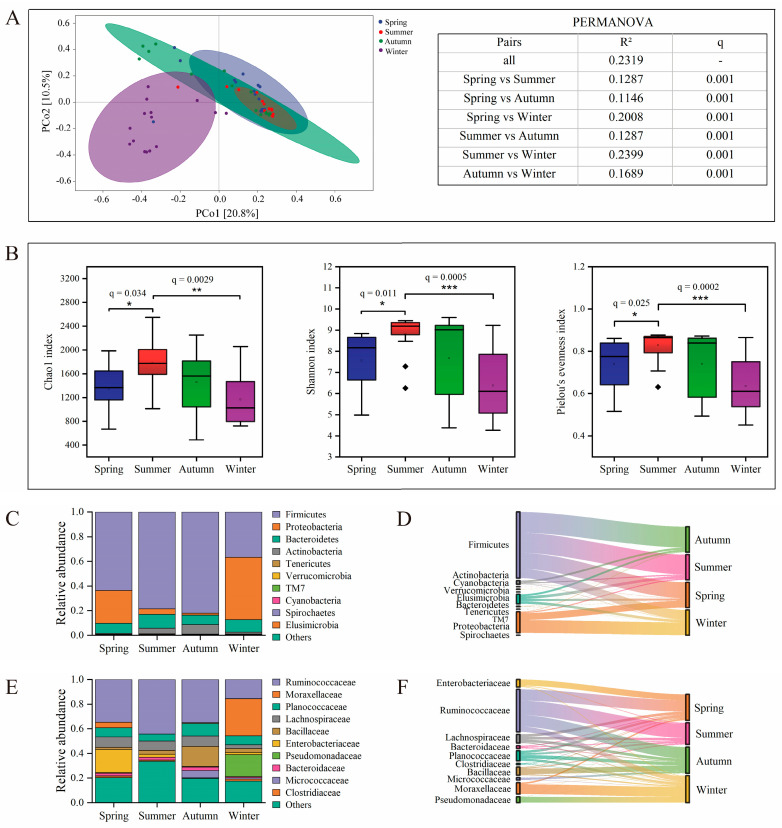
Seasonal variation in the gut microbiota composition and diversity of sika deer: (**A**) PCoA of ASVs relative abundances, using Bray–Curtis distances, was performed to visualize compositional differences; each circle represents an individual sample; different colors represent different experimental groups as indicated in the legend. PERMANOVA was applied to test for significant differences in gut microbiota composition among seasons. (**B**) A boxplot of the Chao 1 index, Shannon index, and Pielou’s evenness index illustrates the within–group distribution of the samples from different seasons (medians/IQR); data points falling outside 1.5 times the IQR are defined as outliers and are represented by diamonds. A bar chart shows seasonal differences in the relative abundance of major phyla (**C**) and families (**E**). A Sankey diagram was used to track indicator phyla (**D**) and families (**F**) associated with each season. Lines indicate the relationships between indicator phyla or families and seasons, with colors labeled according to gut microbiota phyla or families; the line width is scaled to reflect the indicator value. Adjusted *p*-values (q) < 0.05 were considered significant and are shown as * q < 0.05, ** q < 0.01, *** q < 0.001.

The gut microbiota comprised 2,658,841 high-quality sequences, 2,643,448 non-singleton sequences, and 36,138 ASVs (Table S5). Beta-diversity analysis, utilizing PCoA ordination (PC1: 20.8%, PC2: 10.5%), revealed distinct seasonal clusters (Figure 2A). Confidence ellipses for spring and winter were clearly separated from each other and from summer. However, the overlapping ellipses for spring, summer, and autumn indicated compositional continuity across these warmer seasons. This pattern received statistical support from a PERMANOVA (adonis2, vegan; 999 permutations), confirming significant compositional differences across all seasonal comparisons (q = 0.001) (Figure 2A, Table S6). Notably, the greatest dissimilarities were observed between summer and winter (R^2^ = 0.2399) and between spring and winter (R^2^ = 0.2008).

Alpha diversity displayed distinct seasonal dynamics in the gut microbiota (Figure 2B, Table S7). Analysis of the Chao1 index revealed significant variations in microbial richness across seasons: summer supported significantly higher microbial richness than both spring (q = 0.034) and winter (q = 0.0029). No significant differences were detected among other seasonal pairs in terms of microbial richness. In terms of community diversity and evenness, both the Shannon and Pielou indices demonstrated consistent seasonal patterns. The Shannon index was significantly higher in summer compared to spring (q = 0.011) and winter (q = 0.0005). For Pielou’s evenness index, summer values were significantly higher than those in spring (q = 0.025) and winter (q = 0.0002). Collectively, these results indicate that summer harbored the most abundant, diverse, and evenly structured gut microbial community compared to other seasons, while spring and winter generally exhibited lower levels of microbial diversity, richness, and evenness.

Taxonomic composition varied significantly across seasons at multiple taxonomic levels. Firmicutes was the dominant phylum in all seasons but was particularly indicative of spring, summer, and autumn. In contrast, winter was uniquely marked by a significant increase in Proteobacteria, which became its indicator phylum (Figure 2C,D and Table S8). At the family level, Ruminococcaceae was the core indicator for the warmer seasons (spring, summer, and autumn). During winter, there was a noticeable shift, with a decline in typical fermentative families and a marked increase in Moraxellaceae and Pseudomonadaceae (both Proteobacteria), which emerged as the dominant and indicator taxa (Figure 2E,F and Table S9). In conclusion, the gut microbiota of sika deer demonstrates substantial seasonal variability associated with temporal changes in diet and environment; establishing adaptive significance would require additional host physiological and functional data.

### 3.3. Effects of Environmental Factors on the Seasonal Variation in Gut Microbiota

**Figure 3:**
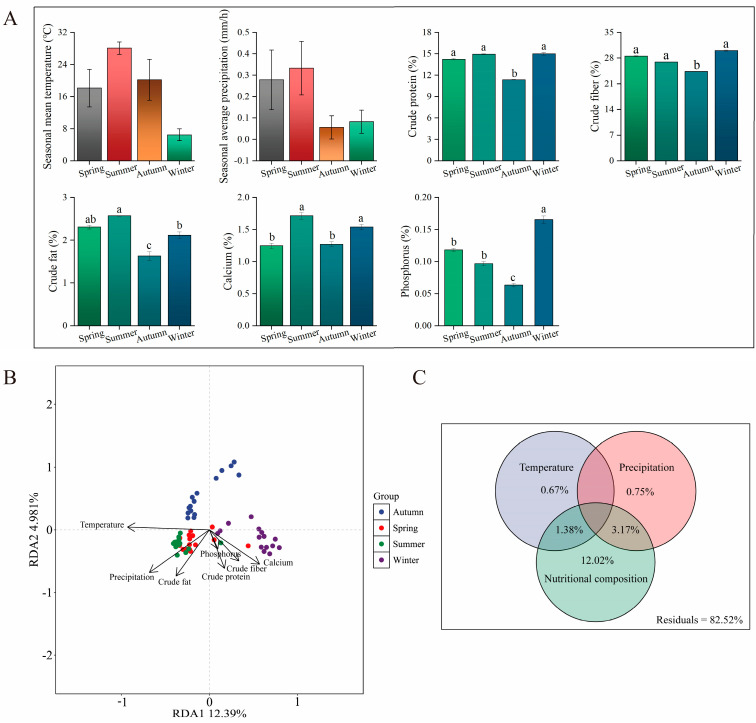
The effects of environmental factors on the seasonal variation in gut microbiota: (**A**) Environmental factors among seasons (Mean ± SD). Bars not sharing a common lowercase letter are significantly different (q < 0.05), as analyzed by the Kruskal–Wallis test followed by Dunn’s post hoc test with Benjamini–Hochberg correction. (**B**) Redundancy analysis (RDA) plots. (**C**) VPA of the three types of factors.

Among the seasons, summer exhibited the highest temperatures and precipitation levels, while winter displayed the lowest temperatures and autumn the least precipitation (Figure 3A). Forage plants demonstrated peak concentrations of crude protein, crude fat, and calcium in summer; the highest levels of crude protein, crude fiber, and phosphorus in winter; and the lowest values for all five nutrients in autumn (Figure 3A, and Tables S10 and S11). An RDA based on ASV tables was performed to assess the effects of environmental factors on gut microbiota (Figure 3B). Both climatic variables (temperature and precipitation) and five dietary components, crude protein, crude fiber, crude fat, calcium, and phosphorus, significantly influenced the seasonal variation in the gut microbiota of sika deer. The vector directions, sample distribution, and Mantel test results collectively supported a significant role of these external factors in shaping the microbial community composition (Table S12). The temperature vector formed acute angles with the samples from spring, summer, and some from autumn, whereas the precipitation vector formed acute angles with samples from spring and summer. Vectors for crude protein, crude fiber, calcium, and phosphorus were closely associated with winter samples. Additionally, VPA using the “varpart” procedure quantified the relative contributions of environmental factors to bacterial community variation (Figure 3C). Temperature, precipitation, and dietary nutrients independently explained 0.67%, 0.75%, and 12.02% of the total variance, respectively. The interaction between temperature and dietary nutrients accounted for 1.38% of the variance, while precipitation combined with dietary nutrients explained 3.17%. VPA indicates that climatic and dietary variables account for only a small fraction of the variation in gut microbiota; most of the variance remains unexplained, likely reflecting unmeasured host and environmental factors, and methodological limitations. To enhance explanatory power and assess causality, future studies should incorporate measurements of host physiology, finer-scale environmental data, and functional omics approaches.

### 3.4. Seasonal Variation in Gut Microbial Function

**Figure 4:**
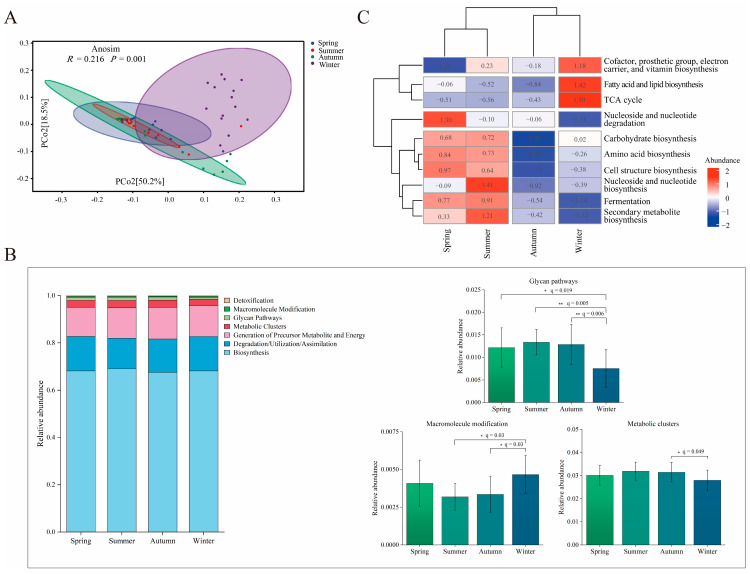
Seasonal variation in gut microbial function: (**A**) A PCoA was performed using Bray–Curtis distances derived from the relative abundance data of Enzyme Commission (EC) units. (**B**) Relative abundance of level–1 MetaCyc pathways (Mean ± SD). Adjusted *p*–values (q) < 0.05 were considered significant and are shown as * q < 0.05, ** q < 0.01. (**C**) Heatmap with cluster analysis of level–2 MetaCyc pathways.

PCoA based on Bray–Curtis distances, calculated from the relative abundance of Enzyme Commission (EC) numbers (inferred from 16S rRNA sequencing data), revealed segregation among individuals across different seasons. Anosim testing revealed significant differences among the four seasonal groups (*R* = 0.216, *p* = 0.001) (Figure 4A). During winter, individuals exhibited the highest enrichment for genes associated with macromolecule modification, displaying significantly higher levels compared to those in summer (q = 0.03) and autumn (q = 0.03). Conversely, individuals in summer and autumn exhibited the greatest enrichment for genes involved in glycan biosynthesis pathways, as evidenced by significantly lower enrichment in winter compared to autumn (q = 0.006), summer (q = 0.005), and spring (q = 0.019). Additionally, genes related to metabolic clusters were most enriched in autumn and summer, with winter showing significantly lower enrichment than autumn (q = 0.049) (Figure 4B, Table S13). A clustering heatmap based on the relative abundance of level-2 MetaCyc pathways indicated that individuals from spring and summer (the growing season) clustered together, while those from autumn and winter (the dormant season) formed a separate cluster (Figure 4C). Notably, pathways related to cofactor, prosthetic group, electron carrier, and vitamin biosynthesis; fatty acid and lipid biosynthesis; and the TCA cycle were significantly enriched in winter individuals compared to those in spring, summer, and autumn.

### 3.5. Seasonal Variation in Major Nutrient Metabolic Pathways

**Figure 5:**
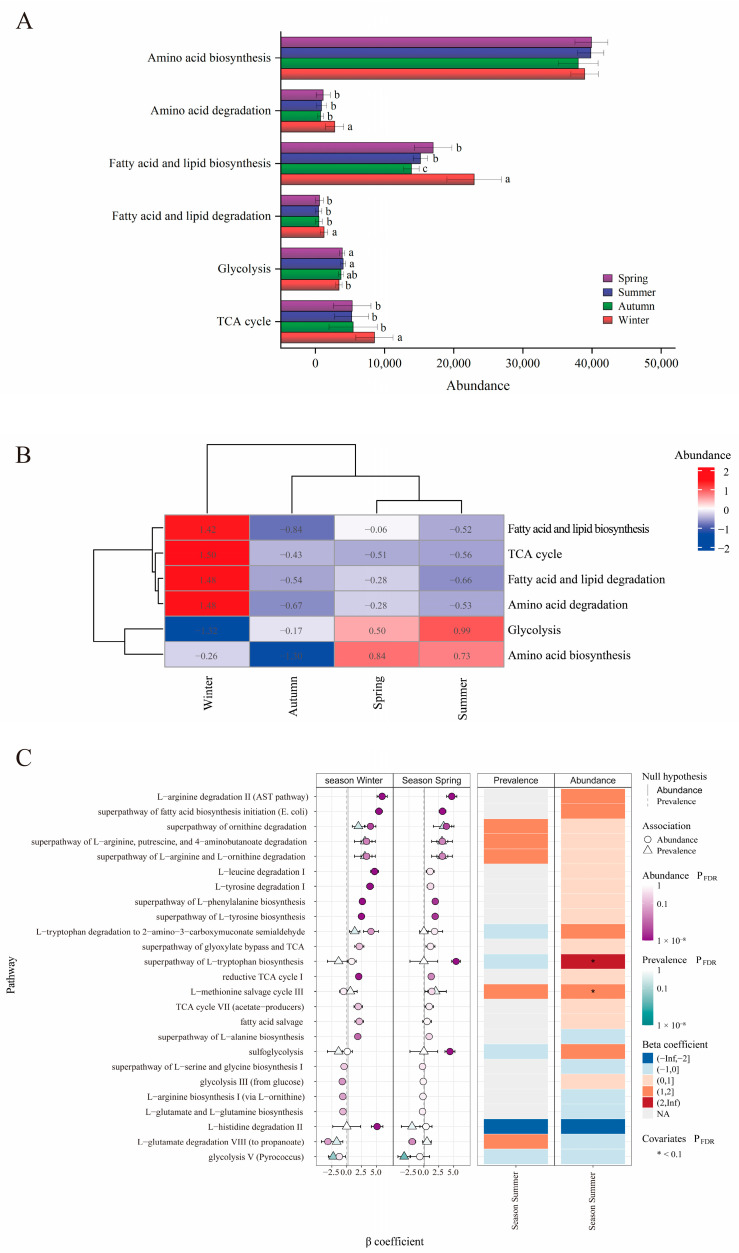
Seasonal variation in major nutrient metabolic pathways: (**A**) Relative abundance of level–2 nutrient metabolic pathways across seasons (Mean ± SD). Bars not sharing a common lowercase letter are significantly different (q < 0.05), as analyzed by the Kruskal–Wallis test followed by Dunn’s post hoc test with Benjamini–Hochberg correction. (**B**) Heatmap with cluster analysis of level–2 nutrient metabolic pathways. (**C**) MaAsLin3 analysis of seasonal differences in the relative abundance of level–3 nutrient metabolic pathways.

The major nutrient metabolic pathways, including amino acid biosynthesis, amino acid degradation, fatty acid and lipid biosynthesis, fatty acid and lipid degradation, glycolysis, and the tricarboxylic acid (TCA) cycle, have been annotated (inferred from 16S rRNA sequencing data). The relative abundances of amino acid degradation, fatty acid and lipid biosynthesis, fatty acid and lipid degradation, and the TCA cycle in winter were significantly higher than those observed in spring, summer, and autumn. Conversely, the relative abundance of the glycolysis pathway in winter was significantly lower than in spring and summer (Figure 5A, Tables S14 and S15). A clustering heatmap based on the relative abundances of these nutrient metabolic pathways showed that individuals from spring, summer, and autumn clustered together, while winter individuals formed a distinct cluster (Figure 5B). These findings are consistent with significant enrichment in the pathways of fatty acid and lipid biosynthesis, fatty acid and lipid degradation, amino acid degradation, and the TCA cycle during winter. In contrast, individuals from spring and summer showed significant enrichment in the glycolysis and amino acid biosynthesis pathways. Meanwhile, individuals from autumn exhibited lower relative abundances across all pathways. Furthermore, MaAsLin3 analysis was conducted to identify level-3 metabolically active pathways (Table S16) significantly enriched in sika deer across different seasons (q < 0.05). This analysis revealed distinct seasonal metabolic signatures (Figure 5C). Winter metabolism was characterized by a dual signature of targeted anabolism and pronounced catabolism. Enriched pathways included catabolic processes such as L-arginine degradation II, superpathway of fatty acid biosynthesis initiation, superpathway of ornithine degradation, superpathway of L-arginine, putrescine, and 4-aminobutanoate degradation, superpathway of L-arginine and L-ornithine degradation, L-leucine degradation I, L-tyrosine degradation I, and L-histidine degradation. Concurrently, anabolic pathways like the reductive TCA cycle, superpathway of L-phenylalanine biosynthesis, superpathway of L-tyrosine biosynthesis, and superpathway of L-alanine biosynthesis were also enriched. This suggests a metabolic state where amino acid catabolism for energy is balanced with the sustained biosynthesis of specific amino acids. Spring exhibited a shift towards amino acid catabolism and sulfur metabolism, with significant enrichment in L-arginine degradation II, superpathway of fatty acid biosynthesis initiation, superpathway of ornithine degradation, and sulfoglycolysis. Interestingly, spring was also marked by the concurrent enrichment of biosynthetic pathways, including the superpathway of L-tryptophan biosynthesis, the superpathway of L-phenylalanine biosynthesis, and the superpathway of L-tyrosine biosynthesis, indicating complex metabolic remodeling during this seasonal transition. Summer individuals displayed a more specific metabolic signature, with enrichment primarily in the superpathway of L-tryptophan biosynthesis and the L-methionine salvage cycle III.

## 4. Discussion

Our year-long investigation into the diet and gut microbiome of the South China sika deer reveals a potential association between seasonal environmental changes, dietary composition, and functional reprogramming of the gut microbial community. Through the application of DNA metabarcoding and 16S rRNA gene sequencing, we have shown that the gut microbiome of the sika deer is not static but rather a dynamic ecosystem, exhibiting seasonally recurrent shifts in both composition and metabolic potential. These shifts likely represent an adaptive mechanism that allows the deer to extract nutrients and energy more effectively from a variable food supply, thus assisting them in coping with the unique challenges each season presents.

### 4.1. The Diet-Microbiome Axis as a Driver of Seasonal Adaptation

The profound influence of diet on the gut microbiome is a well-established principle in microbial ecology [11]. Our findings corroborate this principle, indicating that seasonal changes in the diet of the sika deer are mirrored in the alterations of their gut microbial communities. The transition from a diverse, protein-rich diet in summer, characterized by a relatively greater abundance of Fabaceae, to a more fibrous, lower-quality diet in winter, marked by increased representation of Rosaceae, was associated with changes in microbial diversity and taxonomic composition. This pattern aligns with observations across a broad spectrum of wild animals, ranging from primates [14,17] and other large herbivores [10] to birds [18], underscoring a universal ecological principle. The consistent dominance of Firmicutes and Bacteroidetes throughout the year typifies herbivores, reflecting their essential role in digesting complex plant polysaccharides [10]. However, the seasonal increase in Proteobacteria, often linked with dysbiosis in humans [35,36,37], is notable. Its elevated presence in wild animals during periods of nutritional stress may signify a potential adaptive response, potentially related to specific metabolic requirements necessary during the dormant season.

Our VPA indicated that diet explained 12.02% of the variation in microbial community composition, a larger proportion than that attributed to climatic variables alone. These results suggest that, while temperature and precipitation provide the environmental context, direct nutritional inputs from forage may play a more significant role in structuring the gut microbial assemblage. This interpretation aligns with studies of other cervids that have reported seasonal dietary shifts as a principal influence on gut microbiome composition [20].

### 4.2. Functional Reprogramming for Seasonal Energy Homeostasis

A significant contribution of our research is the provision of insights into the potential functional consequences of seasonal shifts in the microbiome. The metabolic pathways inferred from our data align with a coordinated functional reprogramming of the gut microbiota, which may facilitate the maintenance of host energy balance throughout different seasons.

During the nutrient-deficient winter months, it appears that the gut microbiome reconfigures its metabolic machinery to optimize energy extraction from high-fiber, low-quality forage. Additionally, the utilization of innovative methods such as Near-Infrared Reflectance Spectroscopy (NIRS) to predict nutrient composition in herbivore fecal samples offers complementary insights and enhances the interpretation of diet-microbiome interactions [38]. This reconfiguration is evidenced by a significant enrichment of pathways related to fatty acid and lipid biosynthesis and degradation, amino acid degradation, and the TCA cycle, indicating a metabolic shift toward catabolism and efficient energy production. The upregulation of the TCA cycle suggests an enhanced capacity for cellular respiration, generating ATP from the limited substrates available. Moreover, increased fatty acid metabolism may serve multiple functions, including the production of energy-rich SCFAs for the host and the maintenance of microbial cell membranes under cold conditions. The notable enrichment in amino acid degradation pathways is particularly significant; it implies that during periods of low dietary protein, the host may depend on the microbiome to break down available proteins and recycle nitrogen, potentially synthesizing essential amino acids that are scarce in the diet. This functional shift to enhance the utilization of raw fiber and nutrient recycling during lean periods has also been observed in wild giant pandas [17] and gibbons [16], indicative of a convergent adaptive strategy among herbivores [39,40].

In stark contrast, the growth seasons of spring and summer are characterized by a metabolic profile oriented towards rapid growth and energy utilization. The enrichment of glycolysis pathways reflects the availability of easily digestible carbohydrates from fresh foliage, facilitating rapid energy harvest. Additionally, the enrichment of amino acid biosynthesis pathways, particularly noticeable in summer, corresponds with the host’s anabolic state during a period of abundant, high-protein forage. The microbiome actively contributes to the host’s growth and reproductive efforts by synthesizing a diverse array of essential amino acids. This functional plasticity, where the microbiome transitions from a catabolic, energy-scavenging mode in winter to an anabolic, growth-supporting mode in summer, has been well-documented in other species, such as humans consuming seasonal produce [11] and primates exploiting fruit-rich seasons [14]. Autumn serves as a transitional period in which the microbiome prepares for the impending winter, marked by the biosynthesis of fundamental amino acids, likely aimed at building host reserves.

Notably, because these interpretations derive from pathway profiles inferred from sequence data, they remain provisional and do not provide direct evidence of microbial activity or host metabolic states. Confirmation will require direct functional and host-level measurements.

### 4.3. Implications for Conservation and Wildlife Management

Understanding the complex interplay among diet, microbiome, and host adaptation is crucial for the conservation of endangered wildlife [41,42]. Our findings highlight the paramount importance of preserving habitat diversity, which provides a varied and seasonally appropriate food supply. The resilience of deer is intimately connected to the functional plasticity of their gut microbiomes, which, in turn, rely on the availability of specific forage plants throughout the year [21,22]. Anthropogenic influences, such as habitat fragmentation, land-use change, and climate change-induced alterations in plant phenology, could disrupt this delicate seasonal balance. Such disturbances might result in a mismatch between available forage and the deer’s metabolic requirements, thereby impairing the microbiome’s adaptive capacity and causing nutritional stress, diminished fitness, and population decline.

These findings have implications for wildlife management strategies. In cases where supplementary feeding is considered necessary during particularly harsh winters, it is advisable for managers to select feed formulations that support, rather than disrupt, the winter-adapted gut microbiome. Specifically, feeds that are disproportionately rich in readily digestible simple carbohydrates could be detrimental to microbiota adapted to high-fiber diets and should, therefore, be used cautiously or avoided.

### 4.4. Limitations and Future Directions

While this study provides an exhaustive examination of the seasonal diet-microbiome relationship in sika deer, it is important to acknowledge certain limitations. Firstly, our functional analysis is based on metabolic potential predicted from 16S rRNA gene markers. Although this method is informative, it does not assess real-time microbial activity. Future research employing metatranscriptomics and metaproteomics is essential to verify whether these predicted functional shifts manifest in gene expression and protein synthesis, as demonstrated in recent primate studies [15].

Secondly, our research, similar to most studies in wildlife microbiome science, is correlational [43,44,45]. While the strong, biologically plausible correlations between diet and microbiome function suggest causality, controlled experiments are required to definitively establish this connection. Thirdly, our analysis utilized fecal samples, which primarily reflect the community in the distal gut and may not fully capture microbial dynamics in other gastrointestinal segments.

Future studies should employ multi-omics approaches, including metabolomics, to directly measure the metabolic outputs of the gut microbiome (e.g., SCFAs, secondary metabolites) and correlate these with the host’s physiological state. Longitudinal monitoring of individuals across multiple years would aid in distinguishing consistent seasonal patterns from variability among individuals. Additionally, exploring the roles of other microbial kingdoms, such as fungi and viruses (phages), could uncover further complexities and regulatory mechanisms within this dynamic ecosystem [46].

## 5. Conclusions

Our study demonstrates that the gut microbiome of the South China sika deer is a responsive and functionally plastic community, attuned to predictable seasonal variations. The observed shifts in microbial metabolic pathways, from an anabolic, growth-oriented profile during resource-abundant seasons to a more catabolic, energy-conserving profile during nutrient-scarce winters, are indicative of the microbiome’s potential role in facilitating host physiological adjustments. These correlational findings suggest that the microbiome may serve as a mediator between the host and its fluctuating environment, potentially influencing the health, resilience, and survival of wild populations.

## Data Availability

The raw sequencing data generated in this study have been deposited in the NCBI Sequence Read Archive (SRA) under two BioProject accessions: PRJNA1431770 for the 16S rRNA gene amplicon sequencing data (intestinal microbiota) and PRJNA1431914 for the diet DNA metabarcoding sequencing data. The data will be publicly available at https://www.ncbi.nlm.nih.gov/sra (accessed on 5 February 2026) following the release date (1 July 2026).

## Supplementary Materials

The following supporting information can be downloaded at https://www.mdpi.com/article/10.3390/microorganisms14030674/s1. Table S1: Diet Sequencing Data Processing and ASV Annotation Result Statistics; Table S2: Diet PERMANOVA Results; Table S3: Diet Alpha Diversity Index Statistics; Table S4: Differences in the Relative Abundance of Major Families (top 10) in Diet Across Seasons; Table S5: Gut Microbiota Sequencing Data Processing and ASV Annotation Result Statistics; Table S6: Gut Microbiota PERMANOVA Results; Table S7: Gut Microbiota Alpha Diversity Index Statistics; Table S8: Differences in the Relative Abundance of Major Phyla (top 10) in Gut Microbiota Across Seasons; Table S9: Differences in the Relative Abundance of Major Families (top 10) in Gut Microbiota Across Seasons; Table S10: Kruskal–Wallis Tests for Differences in Five Dietary Components Across Seasons; Table S11: Results of Dunn’s Test with Benjamini–Hochberg Correction for Seasonal Differences in Five Dietary Components; Table S12: Spearman’s Correlations of Environmental Variables with Gut Bacterial Community Structure as Determined by Mantel test; Table S13: MetaCyc Function Annotation Results; Table S14: Kruskal–Wallis Tests for Differences in Major Nutrient Metabolic Pathways; Table S15: Results of Dunn’s Test with Benjamini–Hochberg Correction for Seasonal Differences in Major Nutrient Metabolic Pathways; Table S16: Major Nutrient Metabolic Pathways Annotation Results.
