# Supplementary material for: Dietary Changes Are Associated with Seasonal Restructuring of the Gut Microbiome in Cervus nippon kopschi
Source: Microorganisms. 2026 Mar 16;14(3):674. doi: 10.3390/microorganisms14030674 (PMC13029723; doi:10.3390/microorganisms14030674)
Supplement: Supplementary file 1 [file microorganisms-14-00674-s001.zip › microorganisms-4168875-supplementary.pdf]

Supplementary Files

Supplementary Tables

Table S1. Diet Sequencing Data Processing and ASV Annotation Result Statistics

Sample ID	Clean sequence	Filtered sequence	Effective sequence	High quality sequence	Non-singleton sequence	ASV Num	Group
Spring1	103063	98878	96424	83586	83541	371	Spring
Spring2	113472	108739	106262	103731	103726	192	Spring
Spring3	114563	110062	107401	101992	101984	146	Spring
Spring4	150623	144345	140826	128901	128884	385	Spring
Spring5	112240	107765	105187	103835	103833	135	Spring
Spring6	106122	101556	99212	97339	97335	154	Spring
Spring7	99771	95586	93210	84389	84374	325	Spring
Spring8	102180	97856	95510	92944	92941	164	Spring
Spring9	92176	88555	86725	85568	85567	164	Spring
Spring10	103680	99326	96976	94286	94286	120	Spring
Spring11	109957	105035	102525	97892	97887	194	Spring
Spring12	110173	105312	102589	96412	96403	237	Spring
Spring13	117587	112472	109734	104180	104158	233	Spring
Spring14	123990	117948	115063	113876	113873	134	Spring
Spring15	132560	126448	123061	116420	116407	224	Spring
Summer1	135636	129682	126496	124153	124151	189	Summer
Summer2	137409	131812	128709	125234	125221	264	Summer
Summer3	127492	121938	118996	116415	116411	158	Summer
Summer4	148468	142136	138936	132126	132112	365	Summer
Summer5	111574	106544	103938	100435	100432	265	Summer
Summer6	136239	130534	127562	121073	121060	363	Summer
Summer7	142110	135709	132507	123543	123512	456	Summer
Summer8	124725	118681	115617	112961	112955	223	Summer
Summer9	143987	137312	133928	125253	125246	360	Summer
Summer10	102864	98722	96496	86812	86782	347	Summer
Summer11	100042	96038	93806	87414	87399	178	Summer
Summer12	98192	94355	92100	88672	88667	202	Summer
Summer13	104082	99897	97514	90373	90365	382	Summer
Summer14	108850	104459	101962	96439	96430	302	Summer
Summer15	113598	109165	106777	100212	100202	331	Summer
Autumn1	101650	97617	95407	89526	89518	326	Autumn
Autumn2	93023	89216	87228	84203	84198	252	Autumn
Autumn3	90292	86627	84637	82705	82696	220	Autumn
Autumn4	89859	86347	84411	81588	81588	230	Autumn
Autumn5	107306	102825	100409	94844	94826	251	Autumn
Autumn6	104850	100592	98316	95598	95588	283	Autumn
Autumn7	61984	59678	58653	55382	55374	256	Autumn

Sample ID	Clean sequence	Filtered sequence	Effective sequence	High quality sequence	Non-singleton sequence	ASV Num	Group
Autumn8	78864	75955	74627	73579	73574	160	Autumn
Autumn9	87658	84393	82926	81374	81366	225	Autumn
Autumn10	87176	84013	82594	80526	80515	208	Autumn
Autumn11	82457	79565	78192	77154	77147	172	Autumn
Autumn12	68381	65941	64868	63800	63799	188	Autumn
Autumn13	125525	122666	121351	117984	117978	210	Autumn
Autumn14	86230	82810	81466	80242	80233	168	Autumn
Autumn15	145255	144764	144092	138893	138885	190	Autumn
Winter1	82821	79595	78187	75784	75781	131	Winter
Winter2	95435	91864	90323	85689	85678	254	Winter
Winter3	93322	89668	88081	86473	86470	134	Winter
Winter4	118480	112901	110020	105385	105379	184	Winter
Winter5	112349	107154	104474	98127	98113	244	Winter
Winter6	114189	108947	106070	99769	99758	171	Winter
Winter7	107036	102194	99550	95763	95753	256	Winter
Winter8	121961	116162	113155	109487	109478	190	Winter
Winter9	118132	112609	109749	105996	105981	169	Winter
Winter10	124519	118129	115123	110605	110594	230	Winter
Winter11	116880	110992	108154	101322	101311	263	Winter
Winter12	126188	120504	117634	112583	112569	235	Winter
Winter13	129575	123601	120516	112721	112695	258	Winter
Winter14	128890	123038	119945	114782	114773	182	Winter
Winter15	130150	123677	120552	113965	113946	273	Winter
Total	6657862	6380911	6236759	5962315	5961708	1937	

Sample ID represents the sample name; Clean sequence is the number of sequences after primer identification and removal; Filtered sequence is the number of sequences after removal of low-quality sequences; Effective sequence is the number of sequences remaining after denoising; High quality sequence is the number of sequences after removal of chimeric; Non-singleton sequence is the number of sequences after removal of singleton; ASV Num is the number of amplicon sequence variants (ASVs). Group refers to seasonal groupings.

Table S2. Diet PERMANOVA Results

Group	Sample size	Permutations	Pseudo–F	R^2	P. value	P. adjusted (q)
all	60	999	14.9425	0.4447	0.001	-
Spring vs Summer	30	999	6.0842	0.1775	0.001	0.001
Spring vs Autumn	30	999	21.4058	0.4327	0.001	0.001
Spring vs Winter	30	999	7.1464	0.2039	0.001	0.001
Summer vs Autumn	30	999	19.1509	0.4059	0.001	0.001
Summer vs Winter	30	999	12.0352	0.3012	0.001	0.001
Autumn vs Winter	30	999	37.9293	0.5768	0.001	0.001

Table S3. Diet Alpha Diversity Index Statistics

Sample ID	Chao1	Shannon	Pielou′s evenness	Group
Spring1	420.692	3.832	0.459	Spring
Spring2	209.669	2.714	0.372	Spring
Spring3	161.343	2.037	0.297	Spring
Spring4	401.058	3.952	0.481	Spring
Spring5	169.345	2.994	0.448	Spring
Spring6	179.637	2.834	0.408	Spring
Spring7	384.202	3.554	0.440	Spring
Spring8	196.788	3.184	0.453	Spring
Spring9	189.767	2.740	0.388	Spring
Spring10	151.081	2.339	0.358	Spring
Spring11	227.859	3.272	0.446	Spring
Spring12	274.932	3.767	0.497	Spring
Spring13	275.473	2.385	0.316	Spring
Spring14	143.578	3.036	0.455	Spring
Spring15	255.419	3.667	0.494	Spring
Summer1	215.448	2.616	0.366	Summer
Summer2	288.466	1.064	0.140	Summer
Summer3	166.410	1.897	0.276	Summer
Summer4	408.295	2.651	0.330	Summer
Summer5	313.270	1.522	0.198	Summer
Summer6	406.186	4.434	0.552	Summer
Summer7	517.494	4.354	0.520	Summer
Summer8	254.427	3.370	0.457	Summer
Summer9	396.424	3.706	0.461	Summer
Summer10	423.946	3.728	0.456	Summer
Summer11	211.131	2.054	0.282	Summer
Summer12	265.480	1.269	0.172	Summer
Summer13	481.253	3.519	0.425	Summer
Summer14	353.013	4.257	0.536	Summer
Summer15	373.820	3.691	0.460	Summer

Sample ID	Chao1	Shannon	Pielou's evenness	Group
Autumn1	403.795	2.390	0.297	Autumn
Autumn2	349.497	1.472	0.192	Autumn
Autumn3	296.765	2.414	0.321	Autumn
Autumn4	331.994	1.091	0.144	Autumn
Autumn5	301.771	2.249	0.297	Autumn
Autumn6	365.761	1.951	0.250	Autumn
Autumn7	366.086	1.832	0.230	Autumn
Autumn8	224.903	2.831	0.397	Autumn
Autumn9	278.763	1.952	0.258	Autumn
Autumn10	279.089	1.698	0.227	Autumn
Autumn11	219.237	1.600	0.222	Autumn
Autumn12	251.510	1.690	0.227	Autumn
Autumn13	229.435	2.205	0.303	Autumn
Autumn14	224.876	1.836	0.257	Autumn
Autumn15	195.880	2.357	0.334	Autumn
Winter1	189.978	1.392	0.204	Winter
Winter2	328.967	1.834	0.238	Winter
Winter3	176.410	2.118	0.311	Winter
Winter4	217.323	2.014	0.282	Winter
Winter5	303.251	2.971	0.394	Winter
Winter6	232.734	2.206	0.314	Winter
Winter7	315.566	2.225	0.292	Winter
Winter8	233.723	2.400	0.337	Winter
Winter9	203.224	2.088	0.298	Winter
Winter10	268.751	3.142	0.424	Winter
Winter11	293.585	1.923	0.249	Winter
Winter12	297.697	3.864	0.518	Winter
Winter13	310.972	4.044	0.533	Winter
Winter14	205.931	2.384	0.335	Winter
Winter15	308.672	2.440	0.319	Winter

Sample ID represents the sample name. Chao1: estimated species richness. Shannon index: measure of community diversity (combining richness and evenness); higher values indicate greater diversity. Pielou's evenness: measure of community evenness, ranging from 0 (dominance) to 1 (complete evenness). Group refers to seasonal groupings.

Table S4 Differences in the Relative Abundance of Major Families (top 10) in Diet Across Seasons

Major family	Relative abundance (Mean ± SD)			
	Spring	Summer	Autumn	Winter
Rosaceae	0.326±0.176	0.143±0.116	0.154±0.157	0.505±0.213
Hamamelidaceae	0.055±0.070	0.036±0.040	0.632±0.172	0.006±0.015
Fabaceae	0.040±0.062	0.280±0.328	0.002±0.002	0.027±0.028
Poaceae	0.074±0.098	0.090±0.088	0.001±0.002	0.024±0.024
Smilacaceae	0.063±0.093	0.080±0.117	0.030±0.032	0.006±0.006
Cyperaceae	0.003±0.009	0.001±0.003	0.001±0.002	0.161±0.178
Fagaceae	0.120±0.151	0.008±0.013	0.002±0.005	0.001±0.003
Moraceae	0.016±0.045	0.016±0.017	0.015±0.052	0.077±0.103
Polygonaceae	0.006±0.018	0.100±0.176	0.001±0.001	0.004±0.008
Lauraceae	0.055±0.082	0.001±0.003	0.002±0.004	0.019±0.064
Others	0.241±0.182	0.245±0.194	0.160±0.100	0.171±0.150

Table S5. Gut Microbiota Sequencing Data Processing and ASV Annotation Result Statistics

Sample ID	Clean sequence	Filtered sequence	Effective sequence	High quality sequence	Non-singleton sequence	ASV Num	Group
Spring1	113184	85253	82220	50899	50635	1438	Spring
Spring2	112350	89713	85551	40894	40660	1584	Spring
Spring3	97589	71120	69817	47626	47483	648	Spring
Spring4	92964	69303	66223	36066	35813	1325	Spring
Spring5	93732	70654	67965	43516	43272	1288	Spring
Spring6	80262	60305	57383	30689	30613	1176	Spring
Spring7	95817	72197	68803	39528	39355	1346	Spring
Spring8	77268	60837	57905	33791	33683	1097	Spring
Spring9	87848	66010	63363	36116	36009	1229	Spring
Spring10	123281	93028	91263	65436	65168	920	Spring
Spring11	77668	58617	55551	27906	27719	1166	Spring
Spring12	124440	92483	88642	47616	47392	1626	Spring
Spring13	115083	85441	83319	52978	52815	828	Spring
Spring14	117379	88022	84338	44814	44453	1831	Spring
Spring15	119194	92606	88018	43246	42874	1868	Spring
Summer1	117319	87762	84162	48251	48132	1642	Summer
Summer2	102823	76649	72400	33796	33578	1502	Summer
Summer3	86854	64596	62070	35282	34884	1309	Summer
Summer4	130915	98195	93088	40645	40396	1825	Summer
Summer5	104914	82557	78694	42206	42069	1447	Summer
Summer6	146248	109594	104548	50808	50485	2064	Summer
Summer7	107635	80668	76348	33321	33141	1537	Summer
Summer8	105333	76450	74575	41182	40905	998	Summer
Summer9	118091	88093	84046	41832	41416	1774	Summer
Summer10	118919	89414	84951	39555	39148	1926	Summer
Summer11	141633	105478	101044	47341	46581	2429	Summer
Summer12	116831	87476	83228	40753	40491	1893	Summer
Summer13	133455	99075	94313	44719	44264	2041	Summer
Summer14	104526	76891	73470	42607	42398	1551	Summer
Summer15	109241	81514	77332	37752	37531	1701	Summer
Autumn1	93206	67745	66598	46568	46505	479	Autumn
Autumn2	114225	87442	85513	65642	65589	534	Autumn
Autumn3	142427	107384	101906	44022	43666	2185	Autumn
Autumn4	127372	91880	90160	50316	49933	1009	Autumn
Autumn5	136499	100806	99146	68178	68061	671	Autumn
Autumn6	128762	95778	91844	50689	50408	1590	Autumn
Autumn7	107969	81146	76926	36544	36175	1884	Autumn
Autumn8	95718	71221	68173	42065	41789	1345	Autumn
Autumn9	107675	80687	76428	32517	32338	1759	Autumn
Autumn10	92465	68397	64742	28552	28341	1523	Autumn

Sample ID	Clean sequence	Filtered sequence	Effective sequence	High quality sequence	Non-singleton sequence	ASV Num	Group
Autumn11	102138	75958	71953	30407	30181	1484	Autumn
Autumn12	115142	85095	82057	53749	53515	1329	Autumn
Autumn13	97200	72895	68767	29302	29114	1567	Autumn
Autumn14	122681	96086	91291	40144	39969	1722	Autumn
Autumn15	128688	95957	91119	40008	39607	2128	Autumn
Winter1	98588	71357	69054	37504	37161	1448	Winter
Winter2	90737	67632	65381	46437	46174	905	Winter
Winter3	91582	67888	65944	43526	43369	759	Winter
Winter4	106059	79422	77006	57501	57325	990	Winter
Winter5	93256	70812	68322	53166	52977	763	Winter
Winter6	84780	63740	61862	33673	33462	760	Winter
Winter7	86768	65051	62450	39433	39176	869	Winter
Winter8	119853	90087	85672	44166	43594	2001	Winter
Winter9	126055	94802	91329	60931	60579	1255	Winter
Winter10	106772	80726	77412	50030	49793	1185	Winter
Winter11	118160	88755	86311	60371	60121	881	Winter
Winter12	100124	75622	71558	33818	33533	1667	Winter
Winter13	131709	99699	94581	45473	45198	1686	Winter
Winter14	126850	95479	93821	72850	72650	710	Winter
Winter15	128999	95964	93178	60088	59782	1181	Winter
Total	6595255	4945514	4745134	2658841	2643448	36138	

Sample ID represents the sample name; Clean sequence is the number of sequences after primer identification and removal; Filtered sequence is the number of sequences after removal of low-quality sequences; Effective sequence is the number of sequences remaining after denoising; High quality sequence is the number of sequences after removal of chimeric; Non-singleton sequence is the number of sequences after removal of singleton; ASV Num is the number of amplicon sequence variants (ASVs). Group refers to seasonal groupings.

Table S6. Gut Microbiota PERMANOVA Results

Group	Sample size	Permutations	Pseudo–F	R^2	P. value	P. adjusted (q)
all	60	999	5.6342	0.2319	0.001	-
Spring vs Summer	30	999	4.1550	0.1287	0.001	0.001
Spring vs Autumn	30	999	3.6049	0.1146	0.001	0.001
Spring vs Winter	30	999	7.0446	0.2008	0.001	0.001
Summer vs Autumn	30	999	4.1243	0.1287	0.001	0.001
Summer vs Winter	30	999	8.8419	0.2399	0.001	0.001
Autumn vs Winter	30	999	5.6862	0.1689	0.001	0.001

Table S7. Gut Microbiota Alpha Diversity Index Statistics

Sample ID	Chao1	Shannon	Pielou′s evenness	Group
Spring1	1486.140	6.641	0.642	Spring
Spring2	1649.720	8.838	0.839	Spring
Spring3	668.208	5.018	0.543	Spring
Spring4	1368.940	7.574	0.734	Spring
Spring5	1383.520	7.221	0.707	Spring
Spring6	1222.910	8.714	0.856	Spring
Spring7	1410.970	7.650	0.742	Spring
Spring8	1161.910	8.436	0.840	Spring
Spring9	1286.880	8.782	0.861	Spring
Spring10	941.108	4.978	0.516	Spring
Spring11	1211.660	8.204	0.806	Spring
Spring12	1694.780	8.176	0.776	Spring
Spring13	850.882	5.985	0.626	Spring
Spring14	1921.520	8.659	0.808	Spring
Spring15	1984.470	8.644	0.805	Spring
Summer1	1695.710	9.190	0.870	Summer
Summer2	1591.660	9.216	0.877	Summer
Summer3	1346.580	7.282	0.707	Summer
Summer4	1888.270	9.373	0.871	Summer
Summer5	1514.100	8.972	0.863	Summer
Summer6	2122.180	9.452	0.869	Summer
Summer7	1604.210	9.185	0.871	Summer
Summer8	1012.240	6.254	0.632	Summer
Summer9	1841.840	8.474	0.793	Summer
Summer10	2007.830	9.362	0.864	Summer
Summer11	2548.650	8.792	0.792	Summer
Summer12	1971.650	9.395	0.870	Summer
Summer13	2115.700	9.237	0.850	Summer
Summer14	1619.670	8.801	0.839	Summer
Summer15	1775.040	9.217	0.865	Summer

Sample ID	Chao1	Shannon	Pielou's evenness	Group
Autumn1	488.222	4.594	0.522	Autumn
Autumn2	537.547	4.376	0.493	Autumn
Autumn3	2250.070	9.595	0.872	Autumn
Autumn4	1041.270	6.254	0.635	Autumn
Autumn5	681.668	4.634	0.505	Autumn
Autumn6	1630.140	8.620	0.820	Autumn
Autumn7	1965.510	9.074	0.839	Autumn
Autumn8	1391.510	6.907	0.671	Autumn
Autumn9	1815.050	9.227	0.858	Autumn
Autumn10	1559.950	9.097	0.861	Autumn
Autumn11	1527.370	9.044	0.860	Autumn
Autumn12	1351.830	5.955	0.583	Autumn
Autumn13	1607.120	9.220	0.870	Autumn
Autumn14	1791.020	9.012	0.845	Autumn
Autumn15	2213.090	9.466	0.862	Autumn
Winter1	1469.090	7.852	0.751	Winter
Winter2	937.784	5.249	0.541	Winter
Winter3	796.394	5.075	0.538	Winter
Winter4	1026.600	5.028	0.515	Winter
Winter5	786.801	4.261	0.451	Winter
Winter6	774.028	5.779	0.606	Winter
Winter7	893.885	6.704	0.691	Winter
Winter8	2058.550	8.829	0.812	Winter
Winter9	1292.450	6.220	0.618	Winter
Winter10	1210.500	6.153	0.610	Winter
Winter11	903.616	5.544	0.576	Winter
Winter12	1718.450	9.226	0.865	Winter
Winter13	1721.040	9.151	0.859	Winter
Winter14	721.671	4.627	0.503	Winter
Winter15	1228.730	6.100	0.611	Winter

Sample ID represents the sample name. Chao1: estimated species richness. Shannon index: measure of community diversity (combining richness and evenness); higher values indicate greater diversity. Pielou's evenness: measure of community evenness, ranging from 0 (dominance) to 1 (complete evenness). Group refers to seasonal groupings.

Table S8 Differences in the Relative Abundance of Major Phyla (top 10) in Gut Microbiota Across Seasons

Major family	Relative abundance (Mean ± SD)			
	Spring	Summer	Autumn	Winter
Firmicutes	0.634±0.226	0.785±0.185	0.820±0.132	0.366±0.260
Proteobacteria	0.268±0.264	0.044±0.079	0.016±0.032	0.506±0.286
Bacteroidetes	0.080±0.046	0.113±0.051	0.078±0.064	0.101±0.072
Actinobacteria	0.009±0.004	0.045±0.134	0.076±0.137	0.015±0.018
Tenericutes	0.005±0.004	0.006±0.003	0.004±0.003	0.005±0.006
Verrucomicrobia	0.001±0.001	0.002±0.003	0.002±0.004	0.000±0.001
TM7	0.000±0.000	0.001±0.001	0.001±0.001	0.001±0.002
Cyanobacteria	0.000±0.001	0.001±0.001	0.001±0.001	0.000±0.000
Spirochaetes	0.000±0.000	0.000±0.000	0.000±0.000	0.001±0.002
Elusimicrobia	0.000±0.000	0.000±0.000	0.000±0.000	0.000±0.000
Others	0.002±0.001	0.003±0.002	0.002±0.001	0.004±0.004

Table S9 Differences in the Relative Abundance of Major Families (top 10) in Gut Microbiota Across Seasons

Major family	Relative abundance (Mean ± SD)			
	Spring	Summer	Autumn	Winter
Ruminococcaceae	0.346±0.192	0.442±0.164	0.350±0.252	0.156±0.154
Moraxellaceae	0.046±0.175	0.000±0.000	0.005±0.018	0.301±0.311
Planococcaceae	0.075±0.112	0.056±0.070	0.103±0.143	0.072±0.134
Lachnospiraceae	0.084±0.062	0.077±0.032	0.083±0.061	0.029±0.037
Bacillaceae	0.017±0.018	0.031±0.036	0.164±0.243	0.034±0.037
Enterobacteriaceae	0.188±0.175	0.023±0.044	0.003±0.004	0.016±0.050
Pseudomonadaceae	0.005±0.013	0.000±0.000	0.003±0.008	0.179±0.220
Bacteroidaceae	0.023±0.017	0.023±0.014	0.026±0.026	0.009±0.012
Micrococcaceae	0.001±0.002	0.002±0.008	0.060±0.134	0.012±0.016
Clostridiaceae	0.012±0.020	0.009±0.004	0.005±0.002	0.015±0.013
Others	0.204±0.077	0.336±0.152	0.197±0.084	0.176±0.111

Table S10 Kruskal-Wallis Tests for Differences in Five Dietary Components Across Seasons

Index	P. value
Crude protein	1.75E-06
Crude fat	3.40E-07
Crude fiber	2.65E-05
Phosphorus	5.65E-08
Calcium	0.00044

Table S11 Results of Dunn's Test with Benjamini-Hochberg Correction for Seasonal Differences in Five Dietary Components

Index	Comparison	P. value	P. adjusted (q)
Crude protein	Autumn - Spring	0.00021	0.00042
Crude protein	Autumn - Summer	1.08E-05	3.23E-05
Crude protein	Spring - Summer	0.48365	0.58039
Crude protein	Autumn - Winter	1.05E-06	6.30E-06
Crude protein	Spring - Winter	0.23747	0.35621
Crude protein	Summer - Winter	0.63059	0.63059
Crude fat	Autumn - Spring	2.09E-05	6.28E-05
Crude fat	Autumn - Summer	5.44E-08	3.27E-07
Crude fat	Spring - Summer	0.23748	0.23748
Crude fat	Autumn - Winter	0.00404	0.00808
Crude fat	Spring - Winter	0.16760	0.20112
Crude fat	Summer - Winter	0.01043	0.01564
Crude fiber	Autumn - Spring	0.00058	0.00175
Crude fiber	Autumn - Summer	0.00559	0.01119
Crude fiber	Spring - Summer	0.50345	0.50345
Crude fiber	Autumn - Winter	2.30E-06	1.38E-05
Crude fiber	Spring - Winter	0.19849	0.23818
Crude fiber	Summer - Winter	0.05059	0.07588
Phosphorus	Autumn - Spring	0.00114	0.00229
Phosphorus	Autumn - Summer	0.03046	0.03655
Phosphorus	Spring - Summer	0.27693	0.27693
Phosphorus	Autumn - Winter	2.89E-09	1.73E-08
Phosphorus	Spring - Winter	0.00721	0.01082
Phosphorus	Summer - Winter	0.00016	0.00048
Calcium	Autumn - Spring	0.98331	0.98331
Calcium	Autumn - Summer	0.00596	0.01193
Calcium	Spring - Summer	0.00636	0.00954
Calcium	Autumn - Winter	0.00123	0.00741
Calcium	Spring - Winter	0.00132	0.00398
Calcium	Summer - Winter	0.63059	0.75671

Table S12 Spearman’s Correlations of Environmental Variables with Gut Bacterial Community Structure as Determined by Mantel test

Variables	Spearman’s r	P. value	P. adjusted (q)
Temperature	0.486	0.001	0.001
Precipitation	0.247	0.001	0.001
Nutrition of composition	0.509	< 0.001	< 0.001

Table S13 MetaCyc Function Annotation Results

Level 1	Level 2	Abundance (Mean ± SD)			
		Spring	Summer	Autumn	Winter
Biosynthesis	Amine and Polyamine Biosynthesis	1018.72±501.46	603.94±335.99	474.68±264.11	1241.99±739.2
Biosynthesis	Amino Acid Biosynthesis	39907.39±2377.98	39808.31±1901.68	38000.07±2888.61	38930.48±1975.7
Biosynthesis	Aminoacyl-tRNA Charging	1537.4±219.17	1591.63±205.23	1486.18±295.77	1380.09±190.3
Biosynthesis	Aromatic Compound Biosynthesis	3133.38±490.22	3263.87±394.37	3051.13±617.02	2796.63±394.09
Biosynthesis	Carbohydrate Biosynthesis	10622.91±1289.34	10644.82±1099.8	9627.84±1943.54	10313.74±1120.61
Biosynthesis	Cell Structure Biosynthesis	9939.53±468.5	9827.82±582.1	9179.37±908.61	9471.91±352.81
Biosynthesis	Cofactor, Prosthetic Group, Electron Carrier, and Vitamin Biosynthesis	30808.15±4646.13	28892.28±3039.69	27373.99±2778.85	35517.38±4531.23
Biosynthesis	Fatty Acid and Lipid Biosynthesis	17021.29±2710.11	15185.9±1025.31	13876.82±1135.17	22952.92±3929.9
Biosynthesis	Metabolic Regulator Biosynthesis	228.86±289.14	52.28±128.09	15.44±31.61	579.34±463.4
Biosynthesis	Nucleoside and Nucleotide Biosynthesis	33177.72±1826.65	34620.23±1272.17	32379.95±1746.6	32889.58±1936.46
Biosynthesis	Other Biosynthesis	355.01±360.62	104.39±165.62	51.02±68.47	1008.46±464.93
Biosynthesis	Secondary Metabolite Biosynthesis	5915.15±458.61	6212.33±148.64	5660.07±568.16	5418.68±386.97
Degradation/Utilization/Assimilation	Alcohol Degradation	118.07±100.03	60.59±67.42	113.91±216.03	147.81±315.16
Degradation/Utilization/Assimilation	Aldehyde Degradation	87.54±86.36	8.63±15.57	3.85±7.56	11.06±10.38
Degradation/Utilization/Assimilation	Amine and Polyamine Degradation	948.12±540.2	565.17±336.02	983.74±819.55	915.3±484.08
Degradation/Utilization/Assimilation	Amino Acid Degradation	1133.22±1026.01	893.76±740.4	759.4±406.62	2791.53±1306.74
Degradation/Utilization/Assimilation	Aromatic Compound Degradation	1138.45±2095.16	493.17±957.77	465.55±836.59	3896.51±2091.73
Degradation/Utilization/Assimilation	C1 Compound Utilization and Assimilation	3751.44±816.15	3572.38±694.6	3882.8±1348.42	3861.97±803.4
Degradation/Utilization/Assimilation	Carbohydrate Degradation	6123.29±1407.69	5738.41±617.4	5555.23±1240.51	4327.71±1815.36
Degradation/Utilization/Assimilation	Carboxylate Degradation	5319.45±1097.08	4508.59±321.7	4703.79±433.02	4326.55±1159.74

Level 1	Level 2	Abundance (Mean ± SD)			
		Spring	Summer	Autumn	Winter
Degradation/Utilization/Assimilation	Chlorinated Compound Degradation	1.01±2.64	0.57±2.16	0.11±0.42	0.17±0.25
Degradation/Utilization/Assimilation	Degradation/Utilization/Assimilation - Other	95.21±285.72	33.93±129.54	13.45±32.36	655.39±387.38
Degradation/Utilization/Assimilation	Fatty Acid and Lipid Degradation	603.79±548.27	466.13±478.58	508.02±481.88	1245.23±545.92
Degradation/Utilization/Assimilation	Inorganic Nutrient Metabolism	1634.7±1061.36	1536.46±983.69	1908.28±1558.6	2584.26±911.76
Degradation/Utilization/Assimilation	Nucleoside and Nucleotide Degradation	5854.38±1237.68	5510.64±597.54	5519.42±960.98	5253.88±1523.93
Degradation/Utilization/Assimilation	Polymeric Compound Degradation	2713.7±910.57	2801.28±664.08	2454.84±1240.92	1774.91±888.57
Degradation/Utilization/Assimilation	Secondary Metabolite Degradation	3165.77±1507.08	2061.43±478.59	2384.54±948.06	2750.29±1380.81
Detoxification	Antibiotic Resistance	853.16±518.96	815.45±457.73	511.25±312.05	852.27±657.91
Detoxification	methanol oxidation to carbon dioxide	5.37±8.47	11.26±43.34	94.58±208.62	42.57±51.77
Generation of Precursor Metabolite and Energy	1,5-anhydrofructose degradation	0.25±0.57	0.72±2.79	0.06±0.23	0.08±0.2
Generation of Precursor Metabolite and Energy	Electron Transfer	1180.23±951.66	1385.34±1024.31	1465.88±1311.62	1890.18±967.04
Generation of Precursor Metabolite and Energy	ethylmalonyl-CoA pathway	2.21±6.38	1.47±2.72	1.3±1.56	0.58±0.7
Generation of Precursor Metabolite and Energy	Fermentation	10266.16±1117.87	10376.32±757.89	9252.89±974.18	8783.83±1559.77
Generation of Precursor Metabolite and Energy	formaldehyde oxidation I	258.94±196.79	244.99±200.27	500.46±534.35	209.08±180.98
Generation of Precursor Metabolite and Energy	Glycolysis	3887.83±384.07	4015±345.3	3711.09±341.48	3411.23±461.84
Generation of Precursor Metabolite and Energy	glyoxylate cycle	475.72±375.83	361.13±343.07	497.99±526.34	799.75±348.05

Level 1	Level 2	Abundance (Mean ± SD)			
		Spring	Summer	Autumn	Winter
Generation of Precursor Metabolite and Energy	isopropanol biosynthesis	2.64±7.52	0.64±1.91	0.43±0.94	4.14±6.53
Generation of Precursor Metabolite and Energy	methyl ketone biosynthesis	6.28±8.53	57.66±222.49	100.04±214.66	39.29±47.18
Generation of Precursor Metabolite and Energy	methylaspartate cycle	3.69±3.94	6.96±14.15	3.3±3.79	1.48±2.3
Generation of Precursor Metabolite and Energy	Pentose Phosphate Pathways	2561.5±312.26	2724.62±107.38	2739.22±87.15	2271.63±356.54
Generation of Precursor Metabolite and Energy	Photosynthesis	1557.63±114.48	1550.28±127.1	1594.63±116.81	1433.21±164.99
Generation of Precursor Metabolite and Energy	Respiration	1196.97±951.84	1417.81±1016.87	1516.03±1282.51	1916.43±966.44
Generation of Precursor Metabolite and Energy	superpathway of glycolysis and Entner-Doudoroff	436.6±241.1	281.43±237.01	449.18±413.02	614.99±305.71
Generation of Precursor Metabolite and Energy	superpathway of glycolysis, pyruvate dehydrogenase, TCA, and glyoxylate bypass	644.58±426.95	555.36±426.55	607.59±577.54	988.81±370.1
Generation of Precursor Metabolite and Energy	TCA cycle	5317.32±2709.93	5243.51±2454.49	5446.78±3481.45	8539.22±2687.6
Glycan Pathways	Glycan Biosynthesis	1477.13±483.36	1630.37±273.16	1565.73±352.64	986.82±458.33
Glycan Pathways	Glycan Degradation	1216.22±375.79	1264.57±249.7	1102.51±520.31	770.05±391.73
Macromolecule Modification	Nucleic Acid Processing	940.97±399.78	703.52±220.45	705.93±260.67	1124.05±333.75
Metabolic Clusters	L-glutamate and L-glutamine biosynthesis	913.6±178.48	892.12±201.52	981.97±127.49	835.34±342.99

Level 1	Level 2	Abundance (Mean ± SD)			
		Spring	Summer	Autumn	Winter
Metabolic Clusters	O-antigen building blocks biosynthesis (E. coli)	1111.21±176.5	1141.02±177.94	944.79±388.37	981.79±211.88
Metabolic Clusters	phospholipases	0±0	0.06±0.22	0±0	0.05±0.19
Metabolic Clusters	pyrimidine deoxyribonucleotide phosphorylation	708.1±235.02	722.43±191.68	738.38±284.66	979.13±245.17
Metabolic Clusters	pyrimidine deoxyribonucleotides biosynthesis from CTP	2.38±2.71	6.11±5.4	14.29±21.53	2.74±5.01
Metabolic Clusters	pyrimidine deoxyribonucleotides de novo biosynthesis I	758.55±262.83	764.69±211.89	716.7±238.68	1061.74±271.64
Metabolic Clusters	pyrimidine deoxyribonucleotides de novo biosynthesis III	357.31±157.09	479.05±73.56	285.49±152.82	236.33±128.62
Metabolic Clusters	pyrimidine deoxyribonucleotides de novo biosynthesis IV	1.67±1.91	4.3±3.83	10.38±15.91	1.93±3.54
Metabolic Clusters	superpathway of L-aspartate and L-asparagine biosynthesis	1345.92±420.28	1349.08±442.17	1367.21±390.49	1149.25±261.79
Metabolic Clusters	tRNA charging	1537.4±219.17	1591.63±205.23	1486.18±295.77	1380.09±190.3

Table S14 Kruskal-Wallis Tests for Differences in Major Nutrient Metabolic Pathways

Index	P. value
Amino Acid Biosynthesis	0.03954
Fatty Acid and Lipid Biosynthesis	1.22E-09
Amino Acid Degradation	0.00079
Fatty Acid and Lipid Degradation	0.00422
Glycolysis	0.00123

Table S15 Results of Dunn's Test with Benjamini-Hochberg Correction for Seasonal Differences in Major Nutrient Metabolic Pathways

Index	Comparison	P. value	P. adjusted (q)
Amino Acid Biosynthesis	Autumn - Spring	0.01175	0.07052
Amino Acid Biosynthesis	Autumn - Summer	0.03382	0.10146
Amino Acid Biosynthesis	Spring - Summer	0.69117	0.69117
Amino Acid Biosynthesis	Autumn - Winter	0.46429	0.55715
Amino Acid Biosynthesis	Spring - Winter	0.07383	0.14766
Amino Acid Biosynthesis	Summer - Winter	0.16440	0.24660
Fatty Acid and Lipid Biosynthesis	Autumn - Spring	4.77E-05	0.00014
Fatty Acid and Lipid Biosynthesis	Autumn - Summer	0.00792	0.01188
Fatty Acid and Lipid Biosynthesis	Spring - Summer	0.15815	0.15815
Fatty Acid and Lipid Biosynthesis	Autumn - Winter	7.37E-11	4.42E-10

Index	Comparison	P. value	P. adjusted (q)
Fatty Acid and Lipid Biosynthesis	Autumn - Spring	0.01443	0.01732
Fatty Acid and Lipid Biosynthesis	Autumn - Summer	0.00011	0.00023
Amino Acid Degradation	Spring - Summer	0.45163	0.67744
Amino Acid Degradation	Autumn - Winter	0.63804	0.76565
Amino Acid Degradation	Spring - Winter	0.77774	0.77774
Amino Acid Degradation	Summer - Winter	0.00022	0.00134
Amino Acid Degradation	Autumn - Spring	0.00330	0.00661
Amino Acid Degradation	Autumn - Summer	0.00128	0.00384
Fatty Acid and Lipid Degradation	Spring - Summer	0.37986	0.56979
Fatty Acid and Lipid Degradation	Autumn - Winter	0.64552	0.77463
Fatty Acid and Lipid Degradation	Spring - Winter	0.67582	0.67582
Fatty Acid and Lipid Degradation	Summer - Winter	0.00088	0.00531
Fatty Acid and Lipid Degradation	Autumn - Spring	0.01443	0.02886
Fatty Acid and Lipid Degradation	Autumn - Summer	0.00417	0.01253
Glycolysis	Spring - Summer	0.19486	0.23383
Glycolysis	Autumn - Winter	0.03382	0.06764
Glycolysis	Spring - Winter	0.40887	0.40887

Index	Comparison	P. value	P. adjusted (q)
Glycolysis	Summer - Winter	0.10971	0.16456
Glycolysis	Autumn - Spring	0.00378	0.01134
Glycolysis	Autumn - Summer	0.00019	0.00118
TCA cycle	Spring - Summer	0.90844	1
TCA cycle	Autumn - Winter	0.82623	1
TCA cycle	Spring - Winter	0.91673	0.91673
TCA cycle	Summer - Winter	0.00365	0.02194
TCA cycle	Autumn - Spring	0.00525	0.01575
TCA cycle	Autumn - Summer	0.00721	0.01443

Table S16 Major Nutrient Metabolic Pathways Annotation Results

Pathway	Annotation	Abundance (Mean ± SD)			
		Spring	Summer	Autumn	Winter
Amino acid biosynthesis	L-methionine salvage cycle III	62.43±43.59	87.37±49.78	28.6±32.6	27.33±26.57
Amino acid biosynthesis	superpathway of L-methionine biosynthesis (transsulfuration)	759.97±362.68	765.36±363.15	746.13±450.07	778.92±250
Amino acid biosynthesis	superpathway of L-alanine biosynthesis	514.55±383.14	237.77±217.68	299.88±357.07	982.13±422.12
Amino acid biosynthesis	superpathway of L-serine and glycine biosynthesis I	1600.72±183.44	1645.09±165.29	1727.43±126.89	1556.87±104.6
Amino acid biosynthesis	superpathway of L-methionine biosynthesis (by sulfhydrylation)	546.23±325.06	427.7±266.17	383.67±238.64	972.78±410.74
Amino acid biosynthesis	L-isoleucine biosynthesis II	1912.22±196.8	1977.37±169.56	1842.55±329.04	1846.16±208.63
Amino acid biosynthesis	L-isoleucine biosynthesis III	1665.11±188.52	1726.63±162.88	1615.47±287.98	1575.37±174.17
Amino acid biosynthesis	L-isoleucine biosynthesis I (from threonine)	1764.15±175.3	1822.55±150.72	1697.25±300.23	1731.26±202.91
Amino acid biosynthesis	L-methionine biosynthesis III	553.4±341.52	513.47±318.81	588.98±418.07	939.67±368.14
Amino acid biosynthesis	superpathway of L-lysine, L-threonine and L-methionine biosynthesis I	807.02±283.08	794.04±236.49	661.43±319.12	916.45±229.97
Amino acid biosynthesis	superpathway of L-phenylalanine biosynthesis	593.19±362.55	234.27±214.67	158±154.77	1061.59±437.94
Amino acid biosynthesis	superpathway of L-threonine biosynthesis	1667.07±243.49	1738.63±197.18	1614.65±325.77	1484.15±226.3
Amino acid biosynthesis	L-histidine biosynthesis	1436.63±193.02	1503.25±153.84	1428.57±235.7	1337.22±155.96
Amino acid biosynthesis	L-lysine biosynthesis III	1638.67±192.82	1703.17±138.17	1612.17±234.28	1464.93±170.96
Amino acid biosynthesis	L-tryptophan biosynthesis	1422.54±142.87	1494.06±117.28	1456.85±126.07	1381.34±139.23
Amino acid biosynthesis	L-arginine biosynthesis I (via L-ornithine)	1411.15±260.46	1492.83±167.67	1438.27±202.89	1134.41±270.5
Amino acid biosynthesis	superpathway of L-tryptophan biosynthesis	412.21±329.59	67.11±112.43	6.27±5.06	18.09±26.27

Pathway	Annotation	Abundance (Mean ± SD)			
		Spring	Summer	Autumn	Winter
Amino acid biosynthesis	superpathway of aromatic amino acid biosynthesis	1687.67±252.23	1753.62±206.28	1644.99±316.46	1511.15±199.51
Amino acid biosynthesis	superpathway of L-aspartate and L-asparagine biosynthesis	1345.92±420.28	1349.08±442.17	1367.21±390.49	1149.25±261.79
Amino acid biosynthesis	L-methionine biosynthesis I	492.92±297.33	517.14±336.15	558.84±417.94	527.82±233.25
Amino acid biosynthesis	superpathway of L-isoleucine biosynthesis I	1689.27±218.33	1756.5±180.92	1636.27±311.15	1551.08±204.93
Amino acid biosynthesis	L-lysine biosynthesis I	1436.38±195.31	1447.84±223.34	1258.08±480.28	1345.32±144.54
Amino acid biosynthesis	L-arginine biosynthesis II (acetyl cycle)	1489.71±180.95	1523.41±159.82	1478.67±187.49	1406.27±118.67
Amino acid biosynthesis	L-valine biosynthesis	1764.15±175.3	1822.55±150.72	1697.25±300.23	1731.26±202.91
Amino acid biosynthesis	L-isoleucine biosynthesis IV	1600.89±540.05	1756.91±422.51	1350.5±806.07	1067.18±530.83
Amino acid biosynthesis	L-lysine biosynthesis VI	1605±199.16	1670.35±145.98	1575.7±246.48	1411.1±185.71
Amino acid biosynthesis	L-glutamate and L-glutamine biosynthesis	913.6±178.48	892.12±201.52	981.97±127.49	835.34±342.99
Amino acid biosynthesis	L-lysine biosynthesis II	423.53±362.86	519.04±376.16	563.74±516.37	355.06±312.91
Amino acid biosynthesis	superpathway of L-tyrosine biosynthesis	576.96±335.94	234.34±215.47	157.07±152.03	929.19±362.13
Amino acid degradation	superpathway of L-arginine, putrescine, and 4-aminobutanoate degradation	24.95±24.63	6.36±12.14	5.87±12.31	34.87±36.95
Amino acid degradation	4-aminobutanoate degradation V	261±193.47	238.65±249.38	306.54±325.43	326.62±216.13
Amino acid degradation	L-arginine degradation II (AST pathway)	128.2±167.99	9.26±14.87	6.59±13.67	210.74±182.99
Amino acid degradation	L-histidine degradation I	374.07±243.3	424.75±230.06	522.08±359.12	690.91±379.78
Amino acid degradation	L-leucine degradation I	153.47±310.99	75.02±219.54	29.23±49.51	674.85±395.42
Amino acid degradation	superpathway of L-arginine and L-ornithine degradation	24.95±24.63	6.36±12.14	5.87±12.31	34.87±36.95
Amino acid degradation	superpathway of ornithine degradation	100.31±96.01	11.43±18.5	6.3±14.06	91.83±77.91
Amino acid degradation	L-glutamate degradation V (via hydroxyglutarate)	32.09±20.83	32.02±15.54	14.17±11.91	60±102.06

Pathway	Annotation	Abundance (Mean ± SD)			
		Spring	Summer	Autumn	Winter
Amino acid degradation	L-histidine degradation II	62.96±168.78	6.03±21.9	10.54±21.1	290.17±256.94
Amino acid degradation	L-glutamate degradation VIII (to propanoate)	0.32±0.52	0.94±0.73	0.61±0.97	0.01±0.05
Amino acid degradation	L-tryptophan degradation to 2-amino-3-carboxymuconate semialdehyde	5.7±13.91	22.02±81.2	0.93±2.75	43.82±83.15
Amino acid degradation	L-tryptophan degradation IX	1.54±5.18	7.26±27.72	0.62±1.96	0.99±1.89
Amino acid degradation	L-tryptophan degradation XII (Geobacillus)	1.39±4.57	6.3±20.06	0.25±0.52	0.61±1.24
Amino acid degradation	L-tyrosine degradation I	107.34±197.4	53.46±109.36	20.95±22.36	446.25±294.87
Amino acid degradation	L-valine degradation I	0.02±0.07	0.05±0.11	1.03±2.16	0.18±0.39
Fatty acid biosynthesis	superpathway of fatty acid biosynthesis initiation (E. coli)	321.04±361.43	96.87±229.48	47.16±87.89	1086.06±491.39
Fatty acid biosynthesis	fatty acid salvage	497.41±577.02	423.79±521.52	472.64±482.17	1050.48±491.69
Fatty acid biosynthesis	fatty acid elongation -- saturated	1176.27±393.14	1191.6±374.12	1050.79±375.14	1594.53±354.75
Fatty acid beta-oxidation	fatty acid β-oxidation I	603.42±548.4	463.35±470.21	507.28±481.69	1113.51±522.48
Glycolysis	glycolysis III (from glucose)	1616.79±278.59	1702.22±191.85	1603.59±282.38	1205.75±367.83
Glycolysis	glycolysis I (from glucose 6-phosphate)	1246.93±151.85	1274.88±179.32	1148.72±218.87	1168.55±109.18
Glycolysis	superpathway of glycolysis and Entner-Doudoroff	436.6±241.1	281.43±237.01	449.18±413.02	614.99±305.71
Glycolysis	glycolysis V (Pyrococcus)	0.08±0.31	1.45±2.83	1.81±2.34	0.3±0.94
Glycolysis	glycolysis II (from fructose 6-phosphate)	1024.04±220.43	1036.45±270.07	956.97±344.13	1036.62±175.2
Glycolysis	sulfoglycolysis	64.48±66.67	6.78±12.12	4.13±8.72	2.35±3.51
TCA cycle	TCA cycle IV (2-oxoglutarate decarboxylase)	693.99±446.32	645.57±426.37	648.06±589.4	1103.53±459.86
TCA cycle	reductive TCA cycle I	458.09±273.09	232.2±84.26	169.28±109.23	890.2±416.45
TCA cycle	incomplete reductive TCA cycle	1052.51±306.67	1137.85±212.91	1026.34±265.99	1052.5±272.82
TCA cycle	reductive TCA cycle II	0±0	0±0	0±0	0.01±0.06

Pathway	Annotation	Abundance (Mean ± SD)			
		Spring	Summer	Autumn	Winter
TCA cycle	TCA cycle VI (obligate autotrophs)	707.43±244.57	726.55±210.91	710.58±283.97	982.96±291.26
TCA cycle	TCA cycle V (2-oxoglutarate:ferredoxin oxidoreductase)	1000.85±304.43	1024.29±280.27	1032.28±389.98	1378.69±297.65
TCA cycle	TCA cycle VII (acetate-producers)	580.82±458.26	471.83±398.97	608.82±605.68	1149.96±476.28
TCA cycle	TCA cycle VIII (helicobacter)	778.23±503.16	944.58±477.59	872.33±638.95	1436.41±486.08
TCA cycle	TCA cycle I (prokaryotic)	985.81±353.25	980.4±300.8	994.96±421.55	1453.65±365.18
TCA cycle	superpathway of glycolysis, pyruvate dehydrogenase, TCA, and glyoxylate bypass	644.58±426.95	555.36±426.55	607.59±577.54	988.81±370.1
TCA cycle	superpathway of glyoxylate bypass and TCA	570.19±463.87	450.3±395.78	579.75±592.83	1034.01±434.47
